# Targeting Cell Signaling and Apoptotic Pathways by Luteolin: Cardioprotective Role in Rat Cardiomyocytes Following Ischemia/Reperfusion

**DOI:** 10.3390/nu4122008

**Published:** 2012-12-12

**Authors:** Tongda Xu, Dongye Li, Dehua Jiang

**Affiliations:** 1 The First Clinical College, Nanjing Traditional Chinese Medicine University, Nanjing, Jiangsu 210046, China; E-Mail: xtd3004@yahoo.com.cn; 2 Research Institute of Cardiovascular Diseases, Xuzhou Medical College, Xuzhou, Jiangsu 221002, China; E-Mail: yiyuanerzhu1021@163.com

**Keywords:** luteolin, ischemia/reperfusion (I/R), ischemia reperfusion injury (IRI), cardiomyocytes, mechanism, apoptosis

## Abstract

Myocardial ischemia often results in damaged heart structure and function, which can be restored through ischemia/reperfusion (I/R) in most cases. However, I/R can exacerbate myocardial ischemia reperfusion injury (IRI). Luteolin, a widely distributed flavonoid, a member of a group of naturally occurring polyphenolic compounds found in many fruits, vegetables and medicinal herbs, has been reported to exhibit anti-inflammatory, antioxidant and anti-carcinogenic activities. In recent years, luteolin has been shown to play an important role in the cardioprotection of IRI. However, its role and mechanism in cardioprotection against IRI has not been clearly elucidated with respect to the apoptosis pathway. The purpose of this paper is to review luteolin’s anti-apoptotic role and mechanism following I/R in rats, and indicate luteolin as a potential candidate for preventing and treating cardiovascular diseases.

## 1. Introduction

Myocardial ischemia refers to a clinical state characterized by low coronary blood flow arising from various causes but resulting in a lack of myocardial oxygen supply which can damage myocardial structure and heart function. In most cases, the damaged structure can be repaired and heart function can be restored to its basal condition through ischemia/reperfusion (I/R) [1,2]. However, in some instances, I/R can have the opposite effect, which not only fails to improve heart function but instead exacerbates cardiac function and worsens structural damage. On the basis of the resultant aggravation in myocardial ischemic tissue damage or even irreversible damage after blood flow is increased, this phenomenon is known as myocardial ischemia reperfusion injury (IRI) [3,4,5]. Therefore, it is necessary for the development of cardioprotective agents to improve myocardial function, decrease the incidence of cardiovascular events and limit the total extent of infarction during I/R. However, earlier pharmacological approaches to attenuate the deleterious effects of IRI have shown limited experimental efficacy or have failed to translate into useful clinical treatments. It is pivotal to keep in mind that a clinical trial is always based on a specific treatment strategy, which may lead to the failure of a drug to achieve its desired effects despite its inherent efficacy. Meanwhile, it is an urgent need to acquire additional insight into the molecular mechanism during I/R, and that could be exploited therapeutically [6]. In addition, such preconditioning effects can occur at relatively low concentrations, while higher levels, which may be difficult to achieve through diet, are needed for direct antioxidant effects [7]. As one of the leading causes of death and disability in industrialized society, cardiovascular diseases need more research, and myocardial IRI which contributes to the morbidity and mortality associated with cardiovascular diseases has become the hot research area. In recent years, emerging research has been shown to possess a remarkable ability to deal with IRI [3,4,5]. Pharmacological preconditioning means the use of drugs to stimulate or simulated endogenous protective substances to decrease IRI. Some drugs have already tested positive for cardioprotective effects through inducing the release of endogenous substances or by directly stimulating endogenous mechanisms to inhibit IRI. The discovery of these major forms of cardioprotective mechanisms has promoted the exploration of new drugs to protect against IRI. Currently, drug pretreatment has been the main area of focus in improving IRI outcomes.

**Figure 1:**
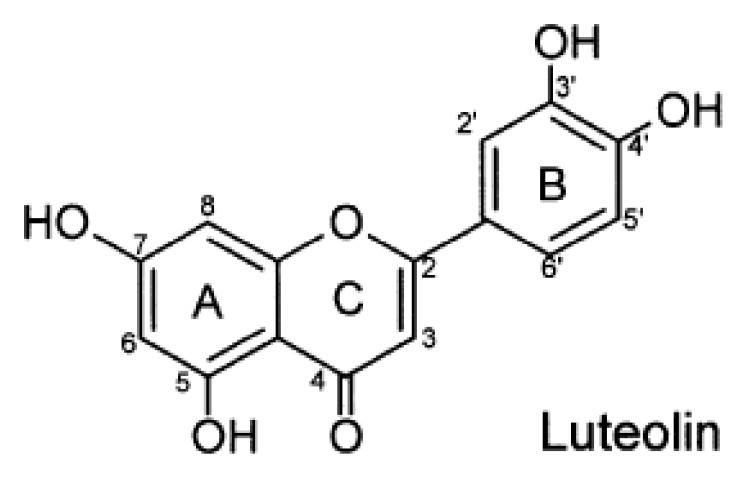
Chemical structure of luteolin.

Luteolin is a widely distributed flavonoid, a member of a group of naturally occurring polyphenolic compounds found in many fruits, vegetables and medicinal herbs and its structure as following (Figure 1) [8]. In recent years, epidemiological evidence suggests that luteolin may play an important role in the decreased risk of acute myocardial infarction (AMI) associated with a diet rich in plant-derived food [9]. Preclinical studies have shown that luteolin possesses a variety of biological and pharmacological activities, including antioxidant, anti-inflammatory, antimicrobial, anticancer, anti-allergic, anti-platelet, and a number of other activities [10,11,12]. In spite of luteolin has been definitely shown to inhibit growth, induce apoptosis, lead to generation of reactive oxygen species and DNA damage in a variety of cancer cells [13]. However, to date, considerable research has indicated that apoptosis is involved in myocardial IRI and that luteolin can play an important anti-apoptotic role during IRI, its role and mechanism in cardioprotection against IRI has not been clearly elucidated with respect to the apoptosis pathway [14,15]. Because previous reviews have already elaborated on the fact that the anti-inflammatory and antioxidant activities of luteolin affect its role in cardioprotection against IRI *in vivo* [10], the aim of this review is to illustrate the underlying mechanism for cardioprotection in anti-apoptosis during I/R by luteolin pretreatment, and clarify the mechanisms by which luteolin regulating apoptosis has revealed new therapeutic targets that could potentially improve heart function in pathologies of myocardial IRI. Moreover, luteolin may be a potential candidate for preventing and treating cardiovascular diseases. In the review paper, the description of inhibition of apoptosis in I/R cardiomyocytes by luteolin is focused on rat cells, rather than other systems. To date, a large number of studies have shown that cardiomyocytes of rat is assessed in I/R by luteolin pretreatment and only cardiomyocytes in other systems such as murine, H9c2, *etc.* [16,17]. Thus, only cardiomyocytes of rat is emphasized in this paper.

## 2. Mechanisms of Cell Apoptosis in Myocardial IRI

To date, some experiments and clinical studies have suggested that cell apoptosis may be an important link during the pathogenesis of myocardial IRI. Fliss and Gattinger observed a typical apoptotic morphological phenotype changed in DNA ladder electrophoresis and reperfusion injury after I/R, indicating that I/R could lead to cardiomyocyte apoptosis in rats [18]. In clinical studies, myocardial apoptosis was also observed in patients with acute coronary syndrome (ACS), even after percutaneous coronary intervention (PCI) treatment. These results further confirmed that ischemia could cause myocardial apoptosis, indicating that cell apoptosis is closely correlated to myocardial IRI. Although concrete mechanisms by which myocardial IRI leads to cellular apoptosis have not been clearly elucidated, a number of potential mechanisms of cell apoptosis in myocardial IRI have been explored, including oxygen free radical, calcium overload and mitochondrial damage. A large quantity of oxygen free radicals are generated during myocardial I/R and promote the development of IRI by lipid and nucleic acid destructive molecular chain reactions. The functional changes in mitochondrial membrane potentially cause the release of apoptosis inducing factor (AIF) from mitochondria or apoptotic protease-activating factor 1 (Apaf-1) in cytoplasm, which in turn activates cysteinyl aspartate-specific proteases (caspase) to induce apoptosis [19]. Moreover, the mitochondria function is significantly changed during I/R, including decreasing mitochondrial membrane potential and energy synthesis. Besides, the cellular Ca^2+^ content increases significantly after the blood flow in ischemic tissues is restored, which causes cellular damage; this process is known as calcium overload. Calcium overload and a series of following harmful metabolic events are the “last common access”. During myocardial I/R, Ca^2+^ is mostly accumulated in the mitochondria, which causes the mitochondrial membrane permeability transition pore (mPTP) to open and facilitates the release of cytochrome c into the cytoplasm, therefore activating caspase to induce apoptosis. All could potentially be used as sites for pharmacotherapy against IRI.

## 3. Luteolin and Signaling Pathways Involved Cell Apoptosis during I/R

**Figure 2:**
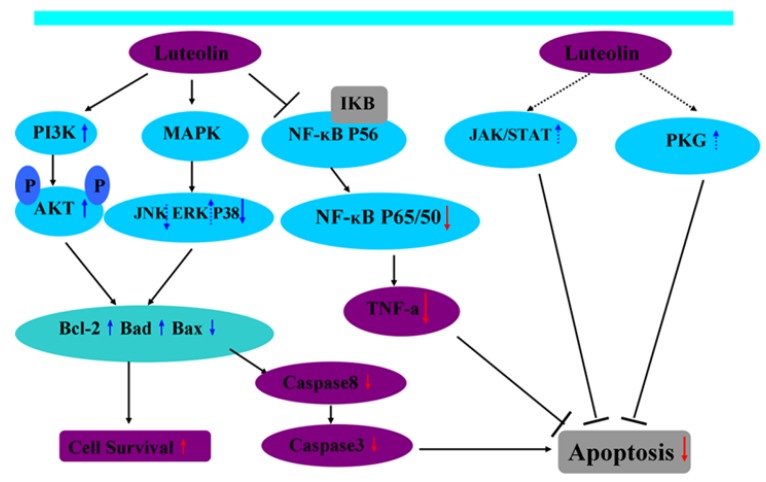
Possible mechanisms for luteolin exerting its protective effects on cardiomyocytes following I/R.

Until now it has been thought that myocardial apoptosis during I/R is mainly involved in the following signaling pathways: phosphatidylinositol-3-kinase/Akt (PI3K/Akt), mitogen-activated protein kinases (MAPKs), caspase, janus kinase/signal transducer and activator of transcription (JAK/STAT), cyclic guanosine monophosphate (cGMP)/protein kinase G (PKG) and lectin-like oxidized low-density lipoprotein receptor-1(LOX-1). By the way, the PI3K/Akt pathway, the MAPKs pathway, and the JAK/STAT pathway are usually known as protective pathways; these pathways are also involved in regulating cell apoptosis. Although the underlying mechanisms of luteolin-induced cardioprotection are complex, they can be summarized as enhancing cell survival by either inhibiting apoptosis or inducing survival signaling in myocardial I/R (Figure 2). 

Pretreatment with luteolin can also upregulate the expression of phosphorylated Akt, which further promotes the expression of Bcl-2 and Bad while downregulating the expression of Bax thereby decreasing apoptosis. Pretreatment with luteolin suppresses NF-κB activation, resulting in decreased expression of NF-κB P65/50 and TNF-α. Meanwhile, the upregulation of Bcl-2 expression can suppress the expression of caspase-8 and caspase-3, resulting in anti-apoptosis [20]. MAPKs are also involved in regulating myocardial IRI. Moreover, JAK/STAT and PKG signaling pathways may mediate cardiomyocytes apoptosis during I/R. 

### 3.1. Luteolin and the PI3K/Akt Signaling Pathway Mediating Cell Apoptosis during I/R

The PI3K/Akt signaling transduction pathway is considered to be the most important signaling pathway involved in controlling cell survival. As a critical regulator of PI3K, Akt can transduce the anti-apoptotic signals in cardiomyocytes, and activated Akt can inhibit cardiomyocyte apoptosis during I/R. It is assumed that the phosphorylation of Akt is down-regulated in myocardial IRI. Recent studies have confirmed the cardioprotective role of this pathway in IRI [21]. *In vivo*, it may be possible to reduce myocardial apoptosis after short periods of ischemia and lessen the infarction area by means of transferring an activated Akt gene to the heart using adenoviral vectors [22]. *In vitro*, the expression of activated PI3K/Akt can reduce hypoxia-induced myocardial apoptosis [23]. We also found that activation of Akt not only reduced mortality in I/R model of cardiomyocytes, but can also improve regional and global cardiac function [24]. Some researchers have studied the interaction between cardiomyocytes positive inotropy and cardioprotection in IRI. In myocardial IRI, damaged cardiomyocytes attenuate contractility and diminish the pump function of the heart. Myocardial positive inotropy means improved cardiomyocytes contractility and results in restored blood supply. Later, apoptosis of cardiomyocytes in IRI is diminished. These findings indicate that Akt signals control the survival and function of cardiomyocytes, but the exact pathway by which this occurs has not yet been fully elucidated. 

In recent years, a large number of experiments have confirmed that the anti-apoptotic effect of luteolin shown in myocardial IRI is related to the activation of PI3K/Akt signaling pathway [24,25,26]. These results indicate that the protective effect of luteolin may occur through the PI3K/Akt signaling pathway. However, the direct cardioprotective effects of luteolin pretreatment on a single cardiomyocyte are still unknown. No previous experiments have investigated the direct effects of luteolin on cardiomyocyte shortening amplitude under I/R conditions. 

Recent results from our research firstly confirmed that luteolin improved I/R-induced cardiomyocyte contractive function, as indicated by the significant dose-dependent increase in single cardiomyocyte shortening amplitude [24,26]. Cardiomyocyte shortening amplitude was indexed as the percentage reduction of cell length after stimulation. Meanwhile, the results demonstrated that luteolin prevented IRI by reducing necrosis and apoptosis in rat cardiomyocytes. We utilized the PI3K inhibitor LY294002 to determine whether the cardioprotective effect of luteolin treatment was indeed mediated by the PI3K/Akt pathway. Inhibition of Akt activity markedly diminished the luteolin-induced positive contraction and inhibition of apoptosis in cardiomyocate following I/R. These results showed that luteolin inhibited apoptosis and improved cardiomyocytes contractile function at least partly through the PI3K/Akt pathway during I/R. 

### 3.2. Luteolin and the MAPKs Signaling Pathway Mediating Cell Apoptosis during I/R

The MAPK signaling pathway is believed to regulate the apoptosis of myocardial cells. MAPKs are serine/threonine protein kinases that are activated by phosphorylation on both a threonine and tyrosine residues. The kinase family has three members, including extracellular signal-regulated kinases (ERK), C-jun *N* terminal kinase/stress-activated protein kinases (JNK/SAPK) and the protein kinase p38. Some researchers have indicated that ERK and p38MAPKs could be activated after myocardial I/R in rats, and other literature has shown that the JNK/SAPK pathway is also involved in the process of myocardial apoptosis during I/R [3,27,28].

Cheng *et al*. reported that luteolin prevented apoptotic neuronal death through reduction in the protein levels of JNK, ERK and p38MAPK and caspase-3 in rat primary cortical cultures [29]. However, whether luteolin can inhibit myocardial apoptosis through the MAPKs pathway during I/R needs to be determined. Recently we tested p38MAPK and phosphorylation of p38MAPK after 3 h simulated ischemia and 2 h simulated reperfusion in cardiomyocytes. Pretreatment with luteolin substantially increased cell viability and shortened amplitude and the expression of phosphorylated p38MAPK was down-regulated [30].

### 3.3. Luteolin and the Caspase Signaling Pathway Mediating Cell Apoptosis during I/R

The caspase signaling pathway includes the mitochondria, death receptor and endoplasmic reticulum (ER) approaches [31]. When apoptotic signals are present, a series of events occur in mitochondria, including mPTP opening, after which the outer membrane ruptures. Therefore, the mitochondrial contents, such as cytochrome C and Apaf-1 are released into the cytoplasm, and the caspase-activating complex, namely the apoptosome forms. Activated caspase-9 in the apoptosome can activate caspase-3 and caspase-7, initiating the caspase cascade to activate a series of proteolytic enzymes in the cell, which eventually results in cellular apoptosis [32]. The death receptor belongs to the tumor necrosis factor (TNF) gene family. TNF and Fas are pro-apoptotic factors that have received much attention in current studies. Binding of Fas ligand to Fas induces the recruitment of procaspase 8 to the receptor complex where the protease becomes activated. Then they form the death induction signal compounds and trigger the caspase cascades, leading to caspase-3 activation and eventually the induction of apoptosis [33]. The ER plays pivotal roles in maintaining Ca^2+^ homeostasis in cells and membrane protein synthesis, modification and folding. Under the stressful condition of myocardial IRI, the ER will induce an unfolded protein response, which eases ER stress by instituting changes in the transcription and translation process, thereby, maintaining cell function. 

**Figure 3:**
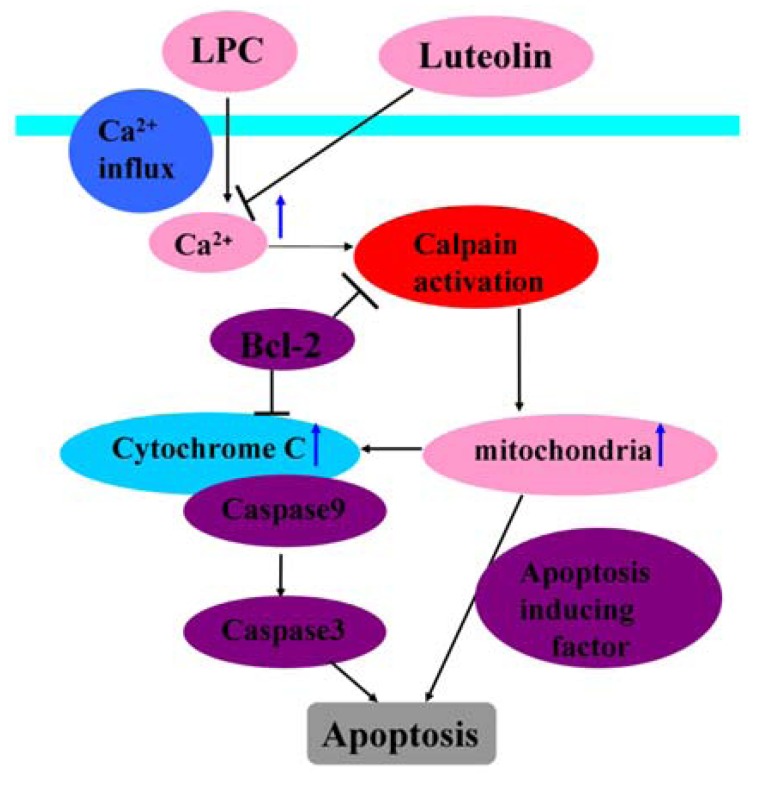
Possible mechanisms of luteolin inhibiting LPC-induced apoptosis in endothelial cells through the blockage of the calcium-dependent mitochondrial pathway.

Song *et al*. found that luteolin inhibits lysophosphatidylcholine (LPC) induced apoptosis in endothelial cells through the blockage of the calcium-dependent mitochondrial pathway [33] (Figure 3). However, whether luteolin inhibits apoptosis in cardiomyocytes during I/R through mediation of the mitochondrial pathway has not been experimentally confirmed. Kim *et al*. investigated whether ER stress and Bcl-2 proteins were linked to the protective effect exerted by luteolin on I/R-induced cardiac damage [16]. The results showed that luteolin pretreatment significantly increased the expression level of the anti-apoptotic protein, Bcl-2, while decreasing that of the pro-apoptotic protein, Bax. Additionally, luteolin down-regulated the expression levels of ER stress proteins. In summary, these results show that the protective effects can be exerted with luteolin pretreatment on I/R-induced cardiac damage by ER approach from caspase signaling pathway. 

LPC-induced apoptosis is characterized by a calcium-dependent mitochondrial pathway involving: calcium influx, activation of calpains, cytochrome C release and caspase activation. Luteolin reduced calcium influx and also inhibited calpain activation and prevented the release of cytochrome C from the mitochondria. The inhibition of cytochrome C release by luteolin blocked activation of caspase-3 and, thus, prevented subsequent cellular apoptosis.

### 3.4. Luteolin and the JAK/STAT Signaling Pathway Mediating Cell Apoptosis during I/R

In recent years, many studies have found that the JAK/STAT pathway is involved in cellar apoptotic processes following I/R in cardiomyocytes [34,35]. Using the JAK-2 specific inhibitor AG490 in a myocardial IRI rat model, it was observed that caspase-3 activity was greatly enhanced, Bax expression was increased and the number of apoptotic cells increased. However, the number of apoptotic cardiomyocytes *in vitro* rat hearts was decreased by AG490. The JAK/STAT pathway potentially has a dual nature in regulating myocardial cellular apoptosis, potentially through crosstalk with another pathway, which means the balance between protective and apoptotic mechanisms is determined by a network of signaling pathways that can interact with the JAK/STAT pathway to regulate the expression of either anti-apoptotic or pro-apoptotic genes [36]. However, whether luteolin inhibits apoptosis in cardiomyocytes, during I/R, via the JAK/STAT pathway has not been experimentally confirmed.

### 3.5. Luteolin and the cGMP/PKG Signaling Pathway Mediating Cell Apoptosis during I/R

cGMP production and PKG activation are common steps in all types of cardioprotection [37]. As an important intracellular messenger substance, cGMP has three main targets, of which PKG is considered the most important target. It has been reported that PKG is involved in the mechanism of preconditioning to protect myocardial cells by activating ATP-sensitive potassium (KATP) channels in myocardial cells [38]. These findings indicate that the cGMP/PKG signaling pathway plays an important role in the regulation of cell apoptosis during I/R. However, whether luteolin inhibits apoptosis in cardiomyocytes during I/R via the cGMP/PKG pathway remains to be elucidated.

### 3.6. Luteolin and the LOX-1 Signaling Pathway Mediating Cell Apoptosis during I/R

Recently, much of the field’s focus has been concentrated on the role of LOX-1 in IRI myocardial cell apoptosis. ROS is able to be released by low density lipoprotein oxidation in I/R. The oxidation of low density lipoprotein and ROS can also cause LOX-1 receptor expression to rise. There were reports that LOX-1 gene expression was up-regulated while cardiac injury was significantly reduced during myocardial I/R [39]. In addition, the research of Kataoka *et al.* has also shown that myocardial I/R can induce LOX-1 expression, however, with the application of LOX-1 monoclonal antibody prior to I/R, the area of myocardial injury has been significantly reduced [40]. These results suggest that LOX-1 can play an important role in I/R-mediated cardiomyocyte apoptosis.

### 3.7. Luteolin and Other Signaling Pathway Mediating Cell Apoptosis during I/R

Besides the above mentioned common signaling pathways, more and more evidence has shown that there are other signaling pathways involved in the myocardial apoptosis during I/R, including NF-κB, small G proteins and protein kinase C (PKC). Despite these signaling pathways seldom being mentioned in the literature, their affect on I/R myocardial apoptosis has been confirmed [20,41,42]. Signaling transduction may involve several signaling pathways, interactions among which form the signaling network. Therefore, one inducer can activate several signaling pathways. Signaling crosstalk means the interaction of two or more different pathways. It is already known that apoptosis is a good target for therapeutic intervention [31].

## 4. Conclusions and Perspectives

At present, a large number of studies have shown that apoptosis plays an important role in myocardial IRI and much work has been undertaken to identify the detailed mechanisms and transduction pathways involved, so that the relationship between cardiomyocyte apoptosis and myocardial IRI can be fully understood. More research has been performed to explore the cardioprotection mechanisms of luteolin treatment during myocardial I/R and some consensus has been obtained that luteolin can exert its anti-apoptotic effect through the PI3K/Akt signaling pathway [24,25,26]. In this way IRI myocardium can be protected, but the effect of luteolin treatment on myocardial IRI protection and the anti-apoptotic influence through the MAPKs signaling pathway and caspase signaling pathway have not yet been completely clarified. Mechanisms for cardiomyocyte protection during myocardial I/R by the JAK/STAT, cGMP/PKG and LOX-1 signaling pathways following luteolin treatment also remain unknown. Moreover, the exact cardioprotective mechanisms of luteolin require detailed illustration, such as the cooperation or crosstalk among PI3K/Akt, MAPKs, cGMP/PKG and JAK/STAT signaling pathways. In summary, luteolin can protect the myocardium against IRI. However, the exact mechanism warrants further investigation.

Some important issues still need to be addressed in future studies: First, most studies so far were performed in cells, not *in vivo*. Moreover, drug treatment in rats needs to be advanced to a more clinically relevant stage. However, there has been little attention to this issue in animal models that are more relevant to physiological and pathological processes *in vivo*. Second, there is a lack of direct evidence supporting the connection between luteolin and some signaling pathways, such as cGMP/PKG, JAK/STAT, and its role in anti-apoptosis during I/R. Therefore, the significance of this potential connection is unclear. In addition, there is the question of possible side effects in cardiac treatment by luteolin pretreatment, as some results have shown that a 20 μg/mL concentration of luteolin has a toxic effect on rat cardiomyocytes in I/R and other adverse effects have not been clearly reported [24,26]. Since the role that these signaling pathways play in luteolin-induced apoptosis has not been fully elucidated, it is still premature to implicate these pathways in this process. In summary, more research is required to clarify the exact effects and mechanisms of luteolin in cardioprotection and apoptosis in cardiomyocytes during I/R. 
